# Seven-step framework to enhance practitioner explanations and parental understandings of research without prior consent in paediatric emergency and critical care trials

**DOI:** 10.1136/emermed-2020-209488

**Published:** 2020-08-29

**Authors:** Louise Roper, Mark D Lyttle, Carrol Gamble, Amy Humphreys, Shrouk Messahel, Elizabeth D Lee, Joanne Noblet, Helen Hickey, Naomi Rainford, Anand Iyer, Richard Appleton, Kerry Woolfall

**Affiliations:** 1 Institute of Population Health, University of Liverpool, Liverpool, UK; 2 Emergency Department, Bristol Royal Children's Hospital, Bristol, UK; 3 Faculty of Health and Applied Sciences, University of the West of England, Bristol, UK; 4 Clinical Trials Research Centre (CTRC), University of Liverpool, Liverpool, UK; 5 Emergency Department, Alder Hey Children's NHS Foundation Trust, Liverpool, Merseyside, UK; 6 Department of Neurology, Alder Hey Children's NHS Foundation Trust, Liverpool, Merseyside, UK

**Keywords:** paediatric emergency med, qualitative research, research, clinical, research, methods

## Abstract

**Background:**

Alternatives to prospective informed consent enable the conduct of paediatric emergency and critical care trials. Research without prior consent (RWPC) involves practitioners approaching parents after an intervention has been given and seeking consent for their child to continue in the trial. As part of an embedded study in the ‘Emergency treatment with Levetiracetam or Phenytoin in Status Epilepticus in children’ (EcLiPSE) trial, we explored how practitioners described the trial and RWPC during recruitment discussions, and how well this information was understood by parents. We aimed to develop a framework to assist trial conversations in future paediatric emergency and critical care trials using RWPC.

**Methods:**

Qualitative methods embedded within the EcLiPSE trial processes, including audiorecorded practitioner–parent trial discussions and telephone interviews with parents. We analysed data using thematic analysis, drawing on the Realpe *et al* (2016) model for recruitment to trials.

**Results:**

We analysed 76 recorded trial discussions and conducted 30 parent telephone interviews. For 19 parents, we had recorded trial discussion and interview data, which were matched for analysis. Parental understanding of the EcLiPSE trial was enhanced when practitioners: provided a comprehensive description of trial aims; explained the reasons for RWPC; discussed uncertainty about which intervention was best; provided a balanced description of trial intervention; provided a clear explanation about randomisation and provided an opportunity for questions. We present a seven-step framework to assist recruitment practice in trials involving RWPC.

**Conclusion:**

This study provides a framework to enhance recruitment practice and parental understanding in paediatric emergency and critical care trials involving RWPC. Further testing of this framework is required.

**Key messages**

**What is already known on this subject**

- Conducting pragmatic randomised controlled trial in paediatric emergency and critical care settings can be challenging, particularly in life-threatening conditions which preclude seeking prospective informed consent for research participation.
- Clinical trials legislation enables children to be entered into a trial without prior informed consent.
- Practitioners may be anxious about approaching families and explaining their child’s research participation without prior consent, although studies have shown how families are supportive of research without prior consent to enable important research.

**What this study adds**

- Recruitment discussions in paediatric emergency and critical care settings are distinct from trials that have time for informed consent discussions.
- This study provides insight into discussions between parents and researchers in a UK emergency medicine-led trial and identifies recruitment practices that facilitate parental understanding of trial purposes and research without prior consent.
- We present a seven-step framework to assist practitioners in discussing future trials with parents who may not be aware that their child has been entered into a clinical trial without their prior informed consent.

## Introduction

Conducting clinical trials in paediatric emergency and critical care settings is challenging for several reasons, primarily over consent. Previously, a key obstacle was the need to administer interventions immediately in life-threatening situations, with no time to seek prospective informed consent from parents for their child’s participation. However, clinical trials legislation^1 2^ enables children to be entered into a trial without prior consent, termed ‘Research Without Prior Consent (RWPC)’. This involves a member of the research team approaching parents after the life-threatening situation has passed. At this point, they are informed of their child’s enrolment into the trial, and consent is sought for continued participation.^3^ Studies have shown parents support RWPC provided they are approached at the appropriate time and the reasons are clearly described as reflected in high consent rates in such trials.^4 5^ Healthcare professionals also support RWPC, even though those unfamiliar with RWPC may be anxious about approaching and discussing this with parents.^6 7^ Guidance on RWPC^8^ has been incorporated into the protocols of the first UK trials to use this process. However, the quality and content of these trial recruitment discussions, as well as parental understanding of practitioner explanations of RWPC, has not yet been explored.

Qualitative research helps understand and optimise recruitment processes in randomised controlled trial (RCTs), including communication between the patient and the enrolling professional.^9 10^ Realpe *et al* 11 analysed recordings of informed consent consultations in a surgical feasibility study (UK FASHIoN- trial) to develop and test a framework for good recruitment practice. Their six-step model included: (1) explaining the medical issue, (2) reassuring the patient about receiving treatment, (3) establishing the uncertainty of the study team, (4) explaining the two arms of the trial, (5) giving a balanced description of treatment strategies and (6) explaining the trial-specific procedures. The model showed potential to enhance recruitment practice in trials seeking informed consent, but is unlikely to be directly transferable to emergency and critical care trials that use RWPC.

We conducted a mixed-methods embedded study in a pragmatic open-label paediatric emergency trial that compared two drugs for the second-line treatment of convulsive status epilepticus, the ‘Emergency treatment with Levetiracetam or Phenytoin in Status Epilepticus in children’ (EcLiPSE), an open label, clinician led trial’.^12^ There was a very low rate of declined consent in this trial (19/404, 4.7%).^13^ We used qualitative methods to explore how practitioners described the trial and RWPC by audiorecording recruitment discussions, and assessed how well this information was understood by interviewing parents within a month of the recruitment discussion. We aimed to use these data to help develop a framework adapted from the Realpe *et al* six-step model,^11^ and specifically designed to optimise recruitment practices and parental understanding of paediatric emergency and critical care trials using RWPC.

## Methods

### Study design, setting and selection of participants

We conducted a mixed-methods embedded study (the Consent study) in all 30 UK sites which enrolled patients into EcLiPSE between July 2015 and April 2017, as reported previously.^6 12 13^ All sites were member sites of Paediatric Emergency Research in the United Kingdom and Ireland (PERUKI,^14^ and composed of both tertiary and secondary level services, with variable levels of experience in enrolling patients into paediatric emergency care trials.

Qualitative methods included audiorecorded trial discussions between parents and practitioners. We conducted telephone interviews with parents approximately 1 month later. Our intention was to explore how practitioners described the trial and RWPC by audiorecording recruitment discussions, and then assessed how well this information was understood by interviewing parents within a month of the recruitment discussion.

Audiorecorded trial discussions usually took place on a hospital ward within 24 hours of randomisation, and verbal and written consent was sought for all data collected. Practitioners were asked to audiorecord all initial and subsequent trial recruitment and consent discussions with parents. LR (female health psychologist) arranged parental telephone interviews via email or telephone with the parent, and explained the consent study aims, objectives and research processes (eg, consent and confidentiality) prior to telephone interview. LR conducted all semistructured interviews using a consent study topic guide (online supplementary file 1) that explored parents’ experiences of recruitment and consent processes, and trial acceptability and conduct. A range of sites were included to ensure sample variance. Sites were closed to consent study recruitment when five interviews had been conducted. Overall recruitment to interviews and recorded trial discussions were stopped when sample variance and data saturation (defined as the point where no new major themes were identified in ongoing analysis) were reached.^15 16^ Interviews and focus groups were transcribed verbatim by a professional transcription company. LR anonymised and checked transcripts for accuracy.

10.1136/emermed-2020-209488.supp1 Supplementary data

### Data analysis

**Figure 1:**
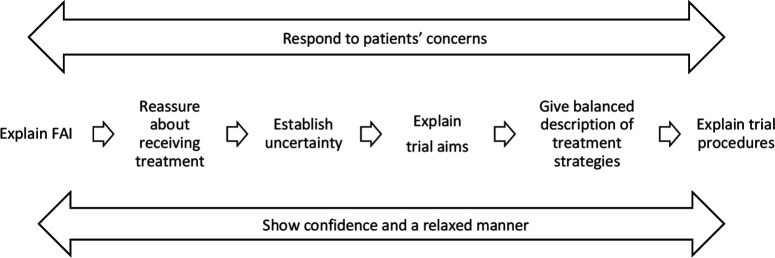
A six step model for recruitment to an RCT (from Realpe *et al*).^11^ FAI, patient condition 'femoroacetabular hip impingement'.

As shown in table 1, data analysis was interpretive and iterative, referring back and forth between developing analysis and gathering new data. We aimed to explore how practitioner trial and RWPC explanations influenced parental understanding, and drew on the six-step model^11^ throughout (figure 1). LR initially analysed data from participants for whom trial discussions and parent telephone interview had been recorded (matched analysis). As part of a thematic approach,^17^ LR and KW (female, social scientist) explored the sequence and content of information provision in trial discussions, as well as the information that was prioritised by parents when they reflected on the recruitment discussion during interviews. These data were compared with matched interviews to determine whether any particular information facilitated parental understanding of the trial and RWPC. NVivo software was used to assist the organisation of qualitative data. We present selected interview quotations (with pseudonyms) that illustrate research themes across a range of participants. Where quotes have been shortened for brevity or to remove identifiable information, omitted text is marked with ‘…’ and explanatory text is in brackets.

**Table 1:** Approach to data analysis and synthesis

Phase	Description
1. Familiarising with qualitative data	Initially two complementary data-coding frameworks were developed for all interview and audiorecorded consent discussion data using broad a priori codes identified from initial reading related to the Consent Study aims and objectives including approach to recruitment and RWPC (please see our linked paper for further details of this stage 1 analysis (add reference to sister paper). Stage 2 analysis was then conducted for the 19 matched audiorecorded consent discussions and parental telephone interview data. LR listened to and read telephone interviews/transcripts and audiorecorded consent discussions/transcripts for each family and noted down initial ideas on themes.
2. Generating initial codes	During the initial data familiarisation stage LR and KW identified data-driven codes and concepts. Analysis was based on a thematic analysis approach, a method for identifying, analysing and reporting patterns (or themes) within data.
3. Developing the coding framework	We then reflected on the Realpe framework and other data driven areas of interest from the stage 1 analysis and initial coding, to help organise matched data framework. We were interested in whether the health professional explained the uncertainty of the trial to the parents, gave the parents a balanced view of the two drugs, explained the study procedures well (eg, randomisation) and responded to questions and answers. LR organised data on each of these aspects of trial recruitment and other data-driven areas of interest. KW second coded 15% (n=3 matched data sets) of transcripts using a framework.
4. Defining and naming themes	Following review and reconciliation, LR and KW revised and ordered themes and related data into steps in an excel spreadsheet. The sequence and content of steps involved consideration what parents prioritised when they described aspects of the trial and the trial discussion during interviews, as well as of how the absence/presence of a particular step in the audiorecorded discussion appeared to hinder/help parental understanding when explored during their interview with LR. Regular weekly or bi weekly meetings were held to discuss the developing framework over a 3-month period.
5. Completion of coding of transcripts	In the final stage of analysis, LR considered the matched analysis framework (and related model) in reference to the wider data set (stage 1), which included an additional 11 interviews. This final stage aimed to ensure that the analysis and related model reflected aspects of recruitment and RWPC discussions that were important to all parents in the sample.
6. Write up	LR and KW developed the initial manuscript. LR led the development of themes and KW developed the final model and manuscript write-up in collaboration with LR and MDL.

RWPC, research without prior consent.

### Patient and public involvement

Parents of children with epilepsy participated in pretrial qualitative research,^18^ which informed the design of EcLiPSE including RWPC processes.^8^ The EcLiPSE trial and embedded consent study team included a patient partner (a parent with relevant experience) as a member of the management team. They helped develop information materials and topic guides for the consent study and dissemination materials, such as an infographic summarising EcLiPSE study findings for participants and members of the public.

## Results

**Figure 2:**
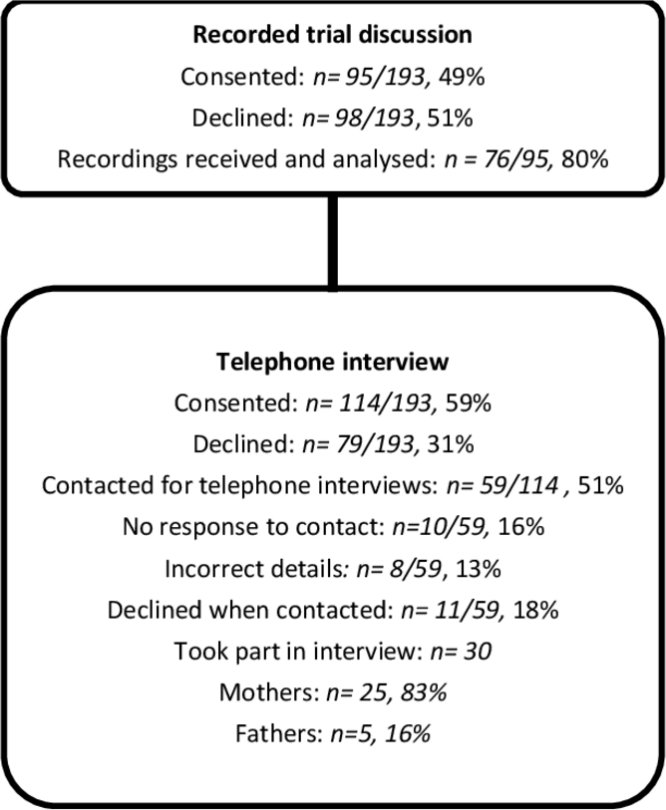
Parent characteristics by method.

A total of 193 children were randomised and treated in the EcLiPSE trial at sites participating in the Consent study. Parents of 95/193 (49%) children gave consent for audiorecorded trial discussions (figure 2), of which 76/95 (80%) were received and analysed. Of the 114 parents who agreed to be approached for interview, 59/114 (51%) were invited, 30 were interviewed and analysed. Of these, 19 were a matched data set (recorded trial discussions and parental interview related to one family) and 11 were not matched. Interview and recorded trial discussions data related to EcLiPSE recruitment at 17/30 (57%) sites.

Just under half (36/76, 47%) of recorded trial discussions were led by doctors and 19/76 (25%) by the research nurses. The practitioner’s clinical background was unclear in 21/76 (27%) recordings. Many (45/76, 60%) recorded one trial discussion, 24/76 (31%) recorded two parts of a trial discussion (eg, part 1 was initial trial information provision and part 2 was consent discussion and form completion a day later) and 7/76 (8%) recorded three or more parts where conversations took place on multiple occasions. In 37 (49%) cases only the second part of the conversation had been recorded.

### Parental capacity

Parental capacity to understand or retain EcLiPSE trial information appeared to be influenced by previous experience of their child having seizures. For example, parents of children who were admitted with their first seizure were often unable to recall the recruitment conversation or describe key aspects of the trial, even when a comprehensive explanation had been provided by practitioners. Poor recall was attributed to the highly emotive situation and often sleep deprivation.

‘I can’t remember…. Yes, because I hardly slept. I stayed in the hospital. I was constantly checking her, so it is really like a big blur to me.’ (Parent interview, mother, P4)

**Figure 3:**
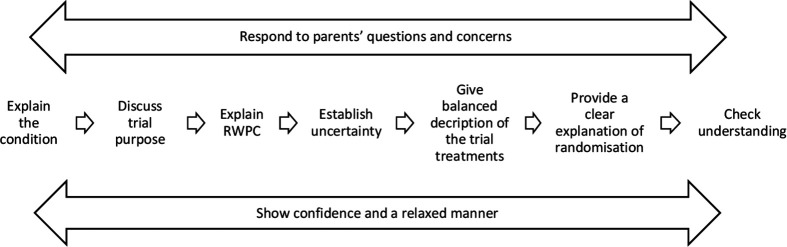
A seven step framework to assist recruitment in trials that involve RWPC. RWPC, research without prior consent.

We found that trial recruitment discussions, including parental understanding of EcLiPSE and use of RWPC, were enhanced when using the seven steps shown in figure 3.

#### Step 1: explain the condition

The first step in the Realpe model (figure 1), ‘explaining the medical condition’, was often not explained or discussed during EcLiPSE discussions. This is likely due to the majority of parents in our sample having prior knowledge of their child’s epilepsy, thereby making an explanation redundant. However, this is not true of trials, and parents need an initial appropriately timed explanation regarding the condition from the clinical or research team. This discussion should include how the condition, or suspected condition, relates to the research question. We have, therefore, placed this as the first step in our framework (figure 3)

#### Step 2: discuss trial purpose

EcLiPSE information posters and leaflets were available for placement in the resuscitation room or emergency department. However, parents reported they were only aware of the trial when they were approached by the recruiting practitioner after the emergency situation had passed. Trial recruitment discussions often began with explanation of the trial aims and objectives and led to improved parental understanding and information retention. The following quote illustrates one mother’s clear recall of the trial purpose 1 month after a detailed description had been provided.

Basically that there's two medicines that they can give that they know stop seizures but they didn’t know which was the best one or the preferred one to use and that’s why you’re doing this study, to see how it affects different people (Parent interview, mother, P15).

In contrast, our analysis showed that brief verbal information provision by the practitioner was linked to poor parental understanding and poor recall of any aspect of the trial, even when information sheets had been provided. For example, one trial discussion (duration 3 min 27 s) briefly covered the trial aims, names of the two drugs and reassurance that their child received ‘the normal treatment she would normally have anyway’ (Recorded trial discussion 2, trial recruiter). When interviewed this parent was unable to recall any details of the conversation: ‘I can't remember’ (Parent interview, mother, P4).

#### Step 3: explain the reasons for conducting research without prior informed consent

Following discussion of the trial’s purpose, practitioners would then explain why informed consent could not have been sought before their child was entered in to the trial:

Obviously a lot of research studies you normally consent before you are entered into a study, but we had to do something called deferred consent because we put her into the study…because of the nature of the emergency department it is not appropriate to try and get consent while she is still fitting (Parent interview, father, P20).

Such explanations were most often well received. Although some parents stated they were initially surprised to discover their child had been enrolled without prior consent, an explanation of the reasons had helped alleviate parental concern.

I think the only thing I really found surprising was the whole informing you about it afterwards…when he explained it all, I mean it makes sense. It’s not the situation to start having discussions, it's an emergency situation (Parent interview, mother, P14).

#### Step 4: establish uncertainty about which treatment is the best

When practitioners explained that they did not know which drug was better (ie, quicker at stopping long-lasting seizures), parents were clear about the uncertainty aspect of the trial; this was not clear when there was no explanation. The following illustrate a good example of this situation:

There are these two drugs, and one of them—we have used both of them for a long time but we have only used one of them in the emergency setting but we are wondering whether the other one might work well, better or worse (Recorded trial discussion 6, Trial recruiter).

What they explained was even though there were two drugs for children with Epilepsy, to try and control it…said they had both been used for years… they just wanted to know which drug would be best suited to control children with epilepsy (Parent interview, mother, P3, matched with recorded trial discussion 6).

#### Step 5: give a balanced description of the trial treatments

A balanced description of both drugs was often closely linked to explanations of uncertainty. Interventions in EcLiPSE were not blinded and therefore staff commonly informed parents which intervention their child had received. They sometimes spoke more positively about the drug received, or did not provide details of the other drug. Although a balanced description was provided in information sheets, tailored practitioner explanations sometimes resulted in parents having an unbalanced understanding of the risks and benefits of the interventions and a potential for misunderstanding. The following example illustrates a parent who was given only minimal information about the allocated treatment. This parent was reassured that her child received the ‘usual treatment’ (phenytoin), yet had no knowledge of the other intervention and therefore did not fully understand the trial aims, treatments or potential risks and benefits of the interventions:

Trial recruiter: It was the old one (your child received), not the new one.

Parent: Okay, yes. The Phenytoin?

Trial recruiter: ‘yes’ (Recorded trial discussion 5)

I think at first when you’re told your daughter is in a trial, you’re like what do you mean she’s in a trial but, at the end of the day, they would have given that medication anyway, so there’s no harm in that. (Parent interview, mother, P8, matched with recorded trial discussion 5).

#### Step 6: provide a clear explanation of randomisation

Practitioners often provided confident but simple descriptions of randomisation, which may have been helped by a tangible nature of the randomisation process, which was the opening of an envelope. When clear explanations were provided, parents were often able to recall accurate information and this was reflected in their use of very similar phrases or terms to those used by the research team:

It’s totally random yes. Now the way that we work is that we’ve got a box of envelopes out the back… all those envelopes are randomly filled beforehand, so we don’t know what we are going to get or anything like that. We open it up, inside is a sheet, and it says on it give this patient (Recorded trial discussion 18, Trial recruiter).

And actually the way that it was explained… we are given the choice of an envelope… but we would’ve given one of those [drugs] anyway (Parent interview, mother, P12).

However, some parents could not recall details of the randomisation process. This reflected a combination of no explanation of randomisation by the research team but also no prior knowledge of randomisation by the parents:

I just know I know what randomised selection and stuff means anyway. I couldn't really tell you how it was explained to me (Parent interview, mother, P27).

#### Step 7: check understanding and provide an opportunity for questions

Parents who gave a very positive description of the trial and who understood key aspects of the trial had developed a good rapport with practitioners. Matched analysis showed these practitioners had longer discussions with parents and encouraged questions. Understanding was further enhanced and retained when parents were asked to describe what they had understood about the purpose of the trial towards the end of the discussion, as illustrated below:

What I have understood is that you have been using this new drug for years, but what you still want to know is the drug still capable of working or is there another way you can control kids who have fits like [child] has…without the research how are you going to know what is the best thing for the kids (Recorded trial discussion 6, parent-matched with parent 3 interview quote shown in step 4).

## Discussion

This qualitative analysis of EcLiPSE trial recruitment and RWPC discussions and subsequent parental interviews, provide new insight into practices which facilitated parental understanding of trial purposes and processes. These data have allowed us to develop a bespoke framework to optimise the process of parent understanding and trial recruitment for future paediatric emergency and critical care trials with RWPC.

While a number of steps from the six-step model^11^ (eg, discussion of the trial’s aims and procedures and giving a balanced description of treatments) were incorporated into trial discussions and facilitated parental understanding in the EcLiPSE trial, the sequence of information presented often differed; there was also the additional step describing RWPC. This sequence is likely to be unique to emergency and critical care trials, as many trial processes have already taken place at the point of a consent discussion.

The first two steps proposed by Realpe *et al*, explanation of the condition and reassurance about receiving treatment, were not always present in our data. This was evident in both sides of the consent discussion, including information provided by practitioners and questions asked by parents. This is likely to reflect parents’ prior knowledge of their child’s condition, making an explanation of seizures unnecessary. However, this is important to consider in future ED trials as parents may not know their child’s diagnosis when the subject is initially raised. In a recent feasibility study^19^ exploring fluid treatment for presumed sepsis in children, some parents were not aware that their child had this diagnosis, and were upset to discover this when approached by the trial recruiter. We, therefore, believe it is an important step and that trial recruiters should check what parents know about their child’s condition and whether further clinical information is needed before broaching research. This first step in our framework (figure 3) is aligned with CONNECT guidance on RWPC^8^ which recommends that research teams should check with clinical staff regarding the child’s condition and parental coping to guide appropriate timing. Discussions should include information about how the condition relates to the research question to enhance parental understanding.

Previous studies have shown that patients and family members often prioritise verbal over written trial information.^4 10 20 21^ This was observed in EcLiPSE, with parents’ descriptions of the trial closely mirroring the phrases and terms used by practitioners rather than the language in the information sheets. Practitioners were flexible in the sequence of information provided, suggesting they tailored the topics with each parent, rather than adhering to the information sheet. Intuitively and as has been shown previously, it is important that trial practitioners tailor discussions to parents’ questions, needs and perspectives.^10 22 23^ Nevertheless, it is important that an explanation of RWPC should be given at an early stage (step 3 in our framework). This will obviate or minimise initial surprise or concern that parents may feel when they first hear about their child’s participation in a trial.^4 5 19^

In contrast to literature suggesting discussions about trial processes, such as randomisation, can be awkward and may be avoided by practitioners,^10 24 25^ practitioners in EcLiPSE gave confident yet simple descriptions of the trial. This likely reflected the EcLiPSE site training, which had enhanced confidence among practitioners.^6^ We believe that descriptions of the randomisation process, which involved the opening of a prefilled envelope, rather than using a benign metaphor (eg, toss of a coin) aided understanding. When such explanations were not provided, parents remained unclear about trials processes as they did not have any prior knowledge about clinical trials methodology to draw on. We believe our seven-step model can be tailored for different trial designs. For example, we propose that discussions about the use of a placebo would logically fall under a description of trial treatments (step 5), while recruitment discussions in a double blind RCT may also include a description of the blinding process (step 6, randomisation).

Finally, our study and proposed framework adds to RWPC literature by highlighting the need for trial recruiters to appropriately time research discussions,^3 8^ conduct them sensitively and provide adequate time for discussion. It also demonstrates the importance of checking parental understanding thereby allowing additional opportunities for practitioners to reiterate aspects of the trial which may not have been clearly explained or fully understood by parents. This seems to be particularly important for parents of children with no previous experience of their child receiving emergency treatment.

### Limitations and future directions

Not all audiorecorded trial discussions could be matched with parent interviews. However, our ‘unmatched’ data corroborated our findings to inform the development of our seven-step framework. We were unable to fully explore one element of Realpe’s model (‘show confidence and a relaxed manner’) as we relied on audiorecordings rather than direct observation. Most parents (75%) of children randomised and treated in EcLiPSE consented to participate in some aspect of the Consent study, and qualitative recruitment stopped when data saturation was reached.^15 16^ However, none of the 19/286 (4%) parents who declined their child’s involvement in EcLiPSE consented to take part in the consent study and therefore their views were not represented in the analysis. Although our framework confirmed a number of steps also shown in the Realpe model, it is an adaptation and requires further testing in future paediatric emergency and critical care trials.

## Conclusions

We have provided a seven-step framework to optimise consent discussions and parental understanding in paediatric emergency and critical care trials. Recruitment discussions for trials in these settings which use RWPC are distinct from trials using prospective informed consent. The proposed framework provides a structured way of delivering trial information, including an explanation of why the research was conducted without prior informed consent. It is important to evaluate this framework in future trials and its impact on recruitment, practitioner confidence in explaining RWPC and parent understanding of trial processes.
