# Supplementary material for: Seven-step framework to enhance practitioner explanations and parental understandings of research without prior consent in paediatric emergency and critical care trials
Source: Emerg Med J. 2020 Aug 29;38(3):198–204. doi: 10.1136/emermed-2020-209488 (PMC7907554; doi:10.1136/emermed-2020-209488)
Supplement: Supplementary data [file emermed-2020-209488supp001.pdf]

Consent study topic guide parent interview example

**Section 1: Demographic information.**	
**Do you mind if I start by asking a few questions about you and your child?**	
1.1	Where do you live?<br>What is your postcode?
	Would you describe yourself as being: employed or unemployed?<br>- (If employed, what is your profession? If Unemployed, what was your previous occupation?)
1.2	How old is your child who was entered into EcLiPSE?
1.3	Is your child a girl or a boy?
1.4	What would you describe as being your first language?
1.5	In which hospital did your child receive treatment in the EcLiPSE trial?
1.6	How long ago was this? *request month and date*?
1.7	Has your child had seizures before this episode?<br>If yes, does your child suffer from a condition which causes seizures? (Explore how long they have had them for, usual treatment, usual frequency, knowledge of the hospital, staff, process of treating seizures)
**Section 2: The EcLiPSE consent process**	
2.1	Would you mind if I start by getting an overall picture of what happened when you first heard about the ECLIPSE trial… could you tell me a bit about that? (Explore any knowledge about the trial before admission)
2.2	During the actual treatment in the room where your child was having the seizure, did you ask any questions about the study? What were you told?
2.3	Did you see any leaflets or posters about the trial?<br>If Yes, where were the posters *(Prompt: on the wall in the ED?)*<br>If Yes, was this before or after the nurse approached you about the trial (Explore initial reactions if before)<br>Could you tell me what you thought about the leaflets/posters?
2.4	Did one of the nursing staff looking after your child introduce you to the research nurse or doctor?
2.5	Did the research nurse/doctor check with you that it was a good time to talk about research?
2.6	Could you tell me what they explained about ECLIPSE?
2.7	Was there anything that you found: a) unclear b) surprising?
2.8	Is there anything else that sticks out in your mind about the discussion?
2.9	How was the nurse/doctor in dealing with you that day?
2.10	Could you tell me about any written information you were given by a nurse or doctor about the trial?
2.11	When did you receive this information *(Prompt: explore written and verbal and time point)*
2.12	Did you read the information leaflet?
2.13	What did you think about the information leaflet?
2.14	Was there anything that you found: a) unclear? b) surprising?
2.15	Could the information leaflet be improved in any way? *(Prompt: If so, how?)*
2.16	**This is a question I ask all parents and it's not a test, but just so we can gage whether the trial is being explained clearly enough I wanted to know whether you**<br>Could you tell me what the ECLIPSE trial was looking at?

2.17	What do you think about the use of deferred consent in an emergency situation (for example, when a child has entered hospital via A&E or born very early)?
2.18	**Explain: Families involved in ECLIPSE provided consent after their child was given the treatment. We call this deferred consent or also known as research without prior consent**<br>Did you know that your child had been given either Keppra or Phenytoin as part of the EcLiPSE trial before the nurse approached you for consent?<br>If NO<br>A) could you tell me how you feel about that now that you know?<br>If YES<br>a) How did the nurse/doctor explain deferred consent to you?<br>b) What did you think when you found out that your child had already been entered into the trial before you were approached by the doctor or nurse about your consent?<br>c) Were you surprised at all? If YES, Could you tell me a bit about that?
2.19	Do you think nurses should briefly discuss the trial with parents in the emergency situation and provide them with the opportunity to saying yes or no to their child's involvement in the trial before the drug has been given?<br>a) Do you think parents would be able to understand such information in this situation?<br>b) Do you think parents might feel under pressure to make a decision in this situation?
2.20	Have you ever heard of the drugs Keppra or Phenytoin before your child's involvement in the EcLiPSE trial?<br>If yes, EXPLORE a) how are you familiar with the drug?<br>b) Do you think that your knowledge of the drug/s influenced your views about the trial? If yes, Explore- how did it influence your views?<br>c) Would you have felt differently about the use of deferred consent if EcLiPSE involved a drug that you were not familiar with?
2.21	Did the nurse/doctor explain to you that blood samples were taken for the trial?<br>If yes…do you know whether blood samples were taken before or after the doctor asked for your deferred consent?<br>If Before: how did you feel about deferred consent for blood samples?
2.23	Children taking part in ECLIPSE were split into two groups by a process called randomisation. Could you tell me if this process was explained to you?<br>How was this process explained?<br>Was there anything about this process that you were unclear about?
2.24	Did you ask any questions about randomisation? (If yes, did this help you make a decision about your child taking part in the trial?)
2.25	Did you ask the nurse/doctor which drug your child had been given?<br>If YES,<br>a) when did you ask this question? (Explore was it before the researcher came to speak to you or during that discussion?)<br>b) how did you feel when they told you which drug your child had been given?<br>c) how would you feel if your child received this drug again in the future?<br>Standard response to question of 'can we have Keppra again'?<br>Emphasise:<br>If the child attends again and needs any second-line treatment and the ED team know the child was randomised then he/she cannot be put into the trial again and should receive the standard drug – phenytoin. If the family want their child to receive Keppra it will be up to the particular ED team medic at that time to decide what to do.
2.26	How long did you get to think about whether you wanted your child's information to be used in ECLIPSE? Do you think this was long enough?

	Did you have the opportunity to ask questions about the study?<br>Did you ask any? (Prompt, what questions did you ask?, if not, why not)<br>How long do you think people should be given to think about taking part in a trial?
2.27	I'd like to ask you now about the questionnaire you were asked to complete at the hospital. Did you complete the questionnaire?
2.28	Do you think it was the right time to complete the questionnaire? (Prompt when would have been a better time?)
2.29	Is there any way you think the questionnaire could have been improved? (Prompt the questions asked, the length of the questionnaire, provided via web link?)
2.30	Is there anything about how ECLIPSE was explained to you that could have been handled a bit differently?
2.31	Did you provide consent for your child to take part in the ECLIPSE trial?
2.32	In making the decision about your child's participation in ECLIPSE, what sort of things went through your mind?
2.33	Some parents have said that it's difficult to take in all the information about a trial when their child is ill. Could you tell me about what it is like to have all this information given to you and for you to think about it at this difficult time
2.34	Could you tell me if you found anything about the trial unclear or confusing?
2.35	Was there anything you found particularly helpful in making up your mind?
2.36	Was there anything you found unhelpful?
2.37	How hard was this decision?
2.38	Was there anything specific that influenced your decision? (Prompt: did you discuss it with anyone who helped or made the decision more difficult?)
2.39	Would you mind telling me what were your reasons for (providing consent/not providing consent)?
**Section 3 Decision making**	
3.4	Did you feel that your child may benefit from taking part in the trial?
3.5	Could you describe the possible benefits you expected your child to gain from taking part in ECLIPSE?
3.6	Did this influence your decision in any way?
3.7	Did you have any concerns about your child taking part?
3.8	What were the possible risks to your child in participating?
3.9	In making your decision, did you think about how the research may benefit other children in the future?
3.10	Apart from the doctor or nurse, did you discuss it with anyone else? [Can you tell me a bit about that?]
3.11	Did you ever feel under pressure in making your mind up? [If yes: where did that pressure come from]?
3.12	Did you know the doctor or nurse a) at the ED b) at the recruitment conversation?
3.13	In your opinion, did your relationship with the doctor or nurse who asked you to take part in ECLIPSE influence your decision? [If so, could you tell me a bit more about that?]
3.14	In making your decision how important was their manor, such as what they said and how they said it?
3.15	Now that a little time has passed, how do you feel about the decision you made?
3.16	Have you thought of any questions you would have liked to have asked that you didn't ask at the time?
**Section 5 Improving the trial and research discussion in the future**	
5.1	When do you think is the best time to approach parents to obtain consent in an emergency situation? (Prompt, ECLIPSE is within 24 hours of admission)

5.2	When do you think parents should be consulted about their child's involvement in an emergency trial [Prompt: what if the trial involved a new drug? Could you tell me a bit more about your reasons for this?]<br>Is deferred consent acceptable for that type of research?
5.3	Who do you think should approach the parents about a trial?<br>Prompt: Do you think it should be a doctor or nurse involved in a child's care who approaches parents about a trial? Do you think it should be someone separate from the care team? *Could you tell me why you think this?*
5.4	Before the ECLIPSE trial, have you ever been approached to consent for your child to participate in medical research? (If yes) [If more than one go through the trial prior to ECLIPSE]<br>*Could you tell me a bit more about it?*<br>*Did you provide consent for your child to take part in the research?*<br>*Could you tell me a bit about what informed your decision (not) to take part?*<br>*Was anyone else involved in making this decision?*<br>*Could you tell me anything about being approached about this research that has stuck in your mind?*
5.5	Do you think that research to improve treatments for critically ill children should be done?
5.6	What advice would you give to researchers who approach families about a clinical trial in an emergency situation?
5.7	Finally, do you feel that taking part in medical research is important? If so, could you tell me why?
**Section 6 Anything else?**	
6.1*	Is there anything else that you think is important to mention that I have not covered?
